# Leptomeningeal Disease and Hydrocephalus as the First Presentation of Melanoma

**DOI:** 10.7759/cureus.44648

**Published:** 2023-09-04

**Authors:** Ahmad Sawalha, Huda Alkilani

**Affiliations:** 1 Neurology, Mayo Clinic, Rochester, USA; 2 General Practice, University of Sharjah, Sharjah, ARE

**Keywords:** melanoma, hydrocephalus, leptomeningeal disease, neurology, acute hydrocephalus, malignant melanoma metastasis, leptomeningeal carcinoma, primary leptomeningeal melanoma

## Abstract

We present a case of an 83-year-old man who developed acute hydrocephalus as the first presentation of leptomeningeal disease secondary to melanoma of unknown primary, which is an exceedingly rare subtype of melanoma, in addition to a very rare complication of malignancy in general, which was diagnosed with imaging as well as cytology modalities. This presentation is rare and highlights the importance of recognizing this condition.

## Introduction

Melanoma of unknown primary is a rare subtype of melanoma, accounting for only 3.2% of total cases of melanoma. Furthermore, primary CNS melanomas are rare, and they constitute about 1% of all cases of melanomas and 0.07% of all brain tumors and are usually encountered in the pediatric population [1,2]. Primary CNS melanoma is thought to be arising from the malignant transformation of melanocytes that are distributed sparsely throughout the leptomeninges [3]. Melanoma involvement of leptomeninges is not associated with any specific clinical or radiological symptoms [4]. The diagnosis of melanoma is mainly dependent on immunohistochemical or immunocytochemical markers, monoclonal antibodies to melanocytic cells (HMB-45), and S-100 protein [5]. The signs and symptoms can be widely variable and will depend primarily on the region involved, which makes the diagnosis challenging, and high clinical suspicion is required. The prognosis of this condition is very poor and it progresses rapidly leading to death in a few days to weeks [6].

## Case presentation

**Figure 1:**
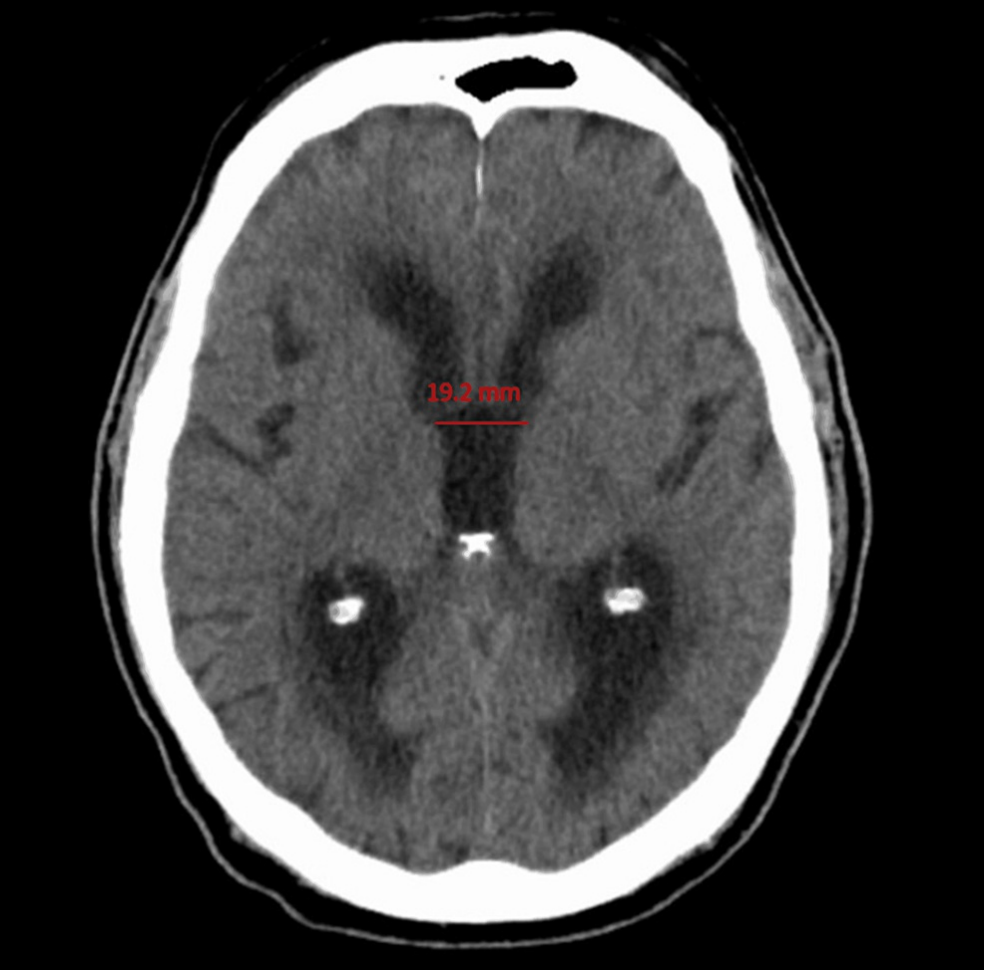
CT of the head without contrast demonstrating increased ventricular size (hydrocephalus) in the lateral and third ventricles.

An 83-year-old male with a past medical history of diabetes mellitus type 2, essential hypertension, and no prior history of malignancy presented with mental and functional decline consisting of acute confusion, magnetic gait, and urinary incontinence over the period of six weeks. The patient had multiple falls, resulting in multiple presentations to the emergency department (ED) due to gait change. CT of the head without contrast at the first ED visit demonstrated increased ventricle size, and the patient was referred to the behavioral neurology clinic for a workup for normal pressure hydrocephalus (NPH) (Figure 1).

**Figure 2:**
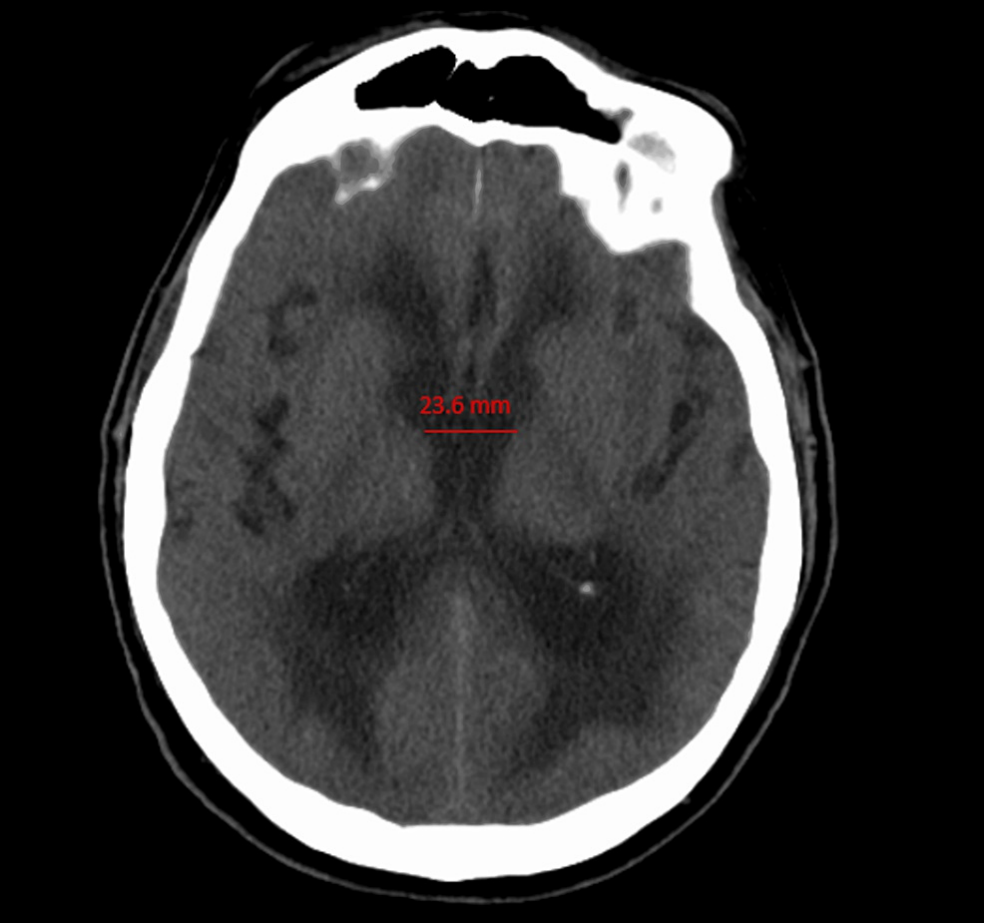
CT of the head without contrast after two weeks from the initial scan shows worsening of hydrocephalus, with increased width of the third ventricle from 19.2 mm to 23.6 mm.

The patient had another fall after two weeks and had a repeat CT of the head that showed a mild increase in the ventriculomegaly as well as loss of sulci within the right frontoparietal region within the right high convexity (Figure 2).

**Figure 3:**
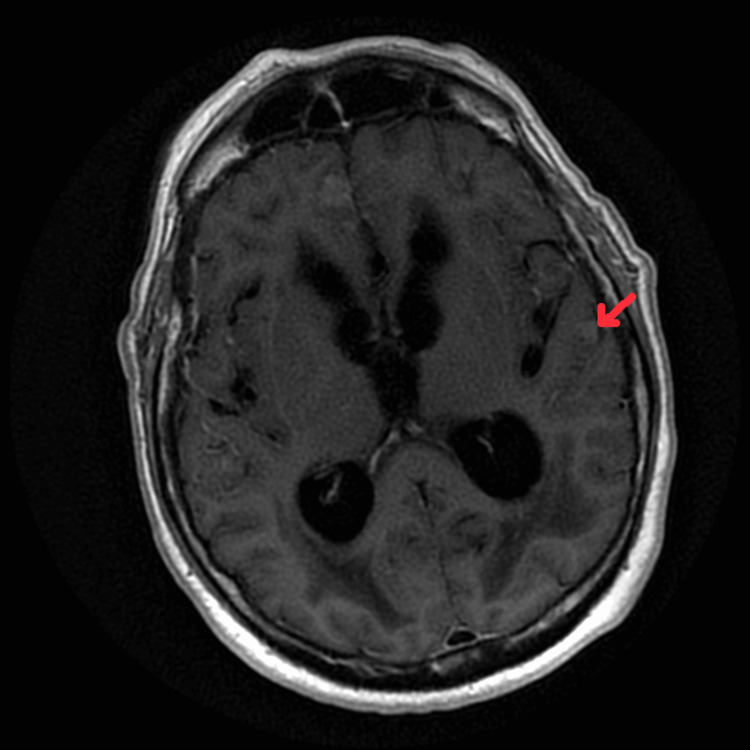
MRI of the brain with and without contrast, non-fat saturated post-contrast T1-weighted image, shows meningeal enhancement within the sulci of the left temporal lobe.

**Figure 4:**
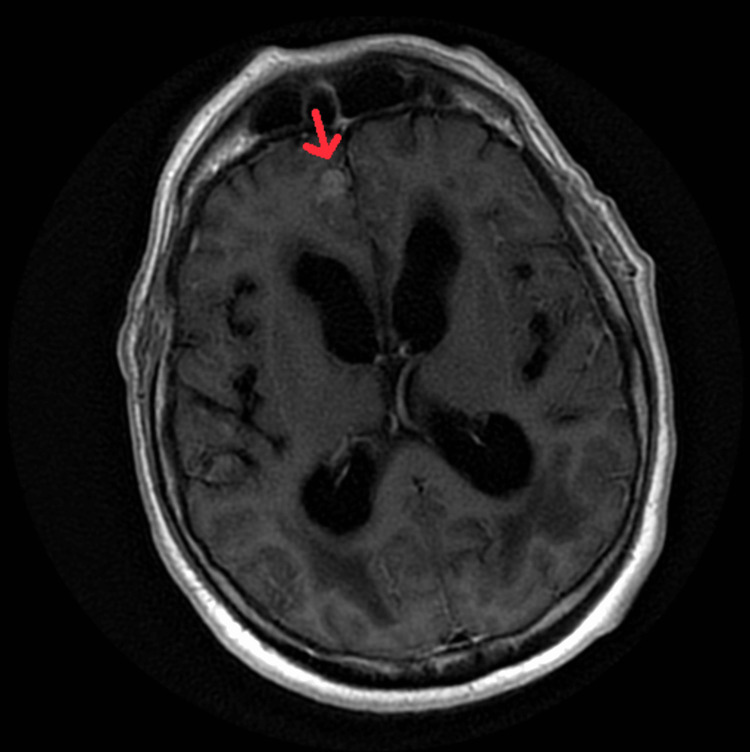
MRI of the brain with and without contrast, non-fat saturated post-contrast T1-weighted image, shows meningeal enhancement within the sulci of the right frontal lobe.

**Figure 5:**
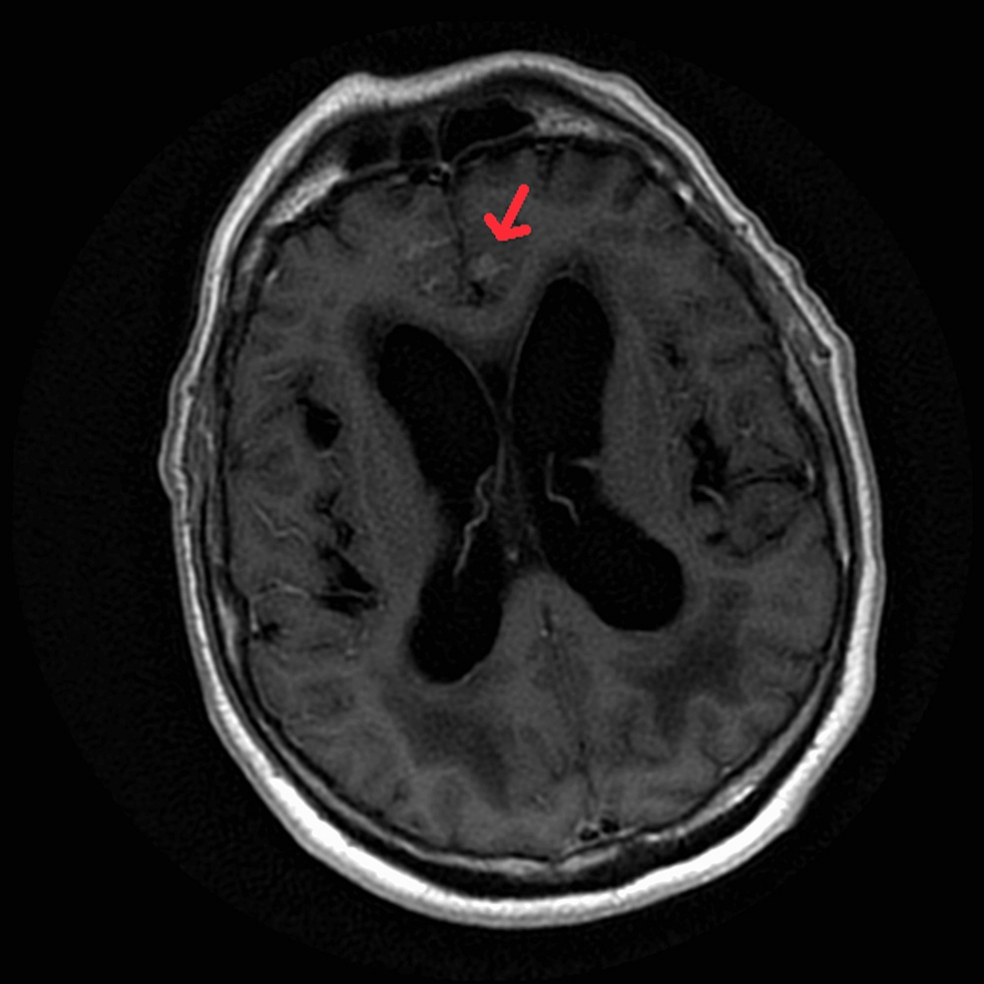
MRI of the brain with and without contrast shows meningeal enhancement within the sulci of the left frontal lobe.

The patient was seen by a behavioral neurologist who suspected NPH and initiated the workup. Lumbar puncture (LP) was scheduled, and the pre-LP Mini-Mental State Examination (MMSE) score was 6. The procedure failed due to technical difficulties, and only 14 cc of CSF was withdrawn. The opening pressure was within normal limits. Analysis of CSF showed low glucose (30 mg/dL), high protein (102 mg/dL), elevated RBCs (79/mm^3^), and normal WBC count (2/mm^3^). Infectious workup came back negative and it included bacterial and fungal cultures, as well as the meningitis encephalitis panel, which is a nucleic acid amplification test (NAAT) of herpes simplex virus (HSV) 1, 2, and 6, varicella-zoster virus (VZV), cytomegalovirus (CMV), *Streptococcus pneumoniae*, streptococcus group B, *Neisseria meningitidis*, *Escherichia coli*, *Listeria monocytogenes*, *Haemophilus influenzae*, and *Cryptococcus neoformans*. Post-LP MMSE was not obtained. The patient was admitted urgently for further workup. Upon admission, the patient had a brain MRI with and without contrast that came back showing abnormal meningeal enhancement within the sulci of the medial frontal lobes and the left temporal lobe (Figures 3-5). Those findings were concerning for leptomeningeal disease.

**Figure 6:**
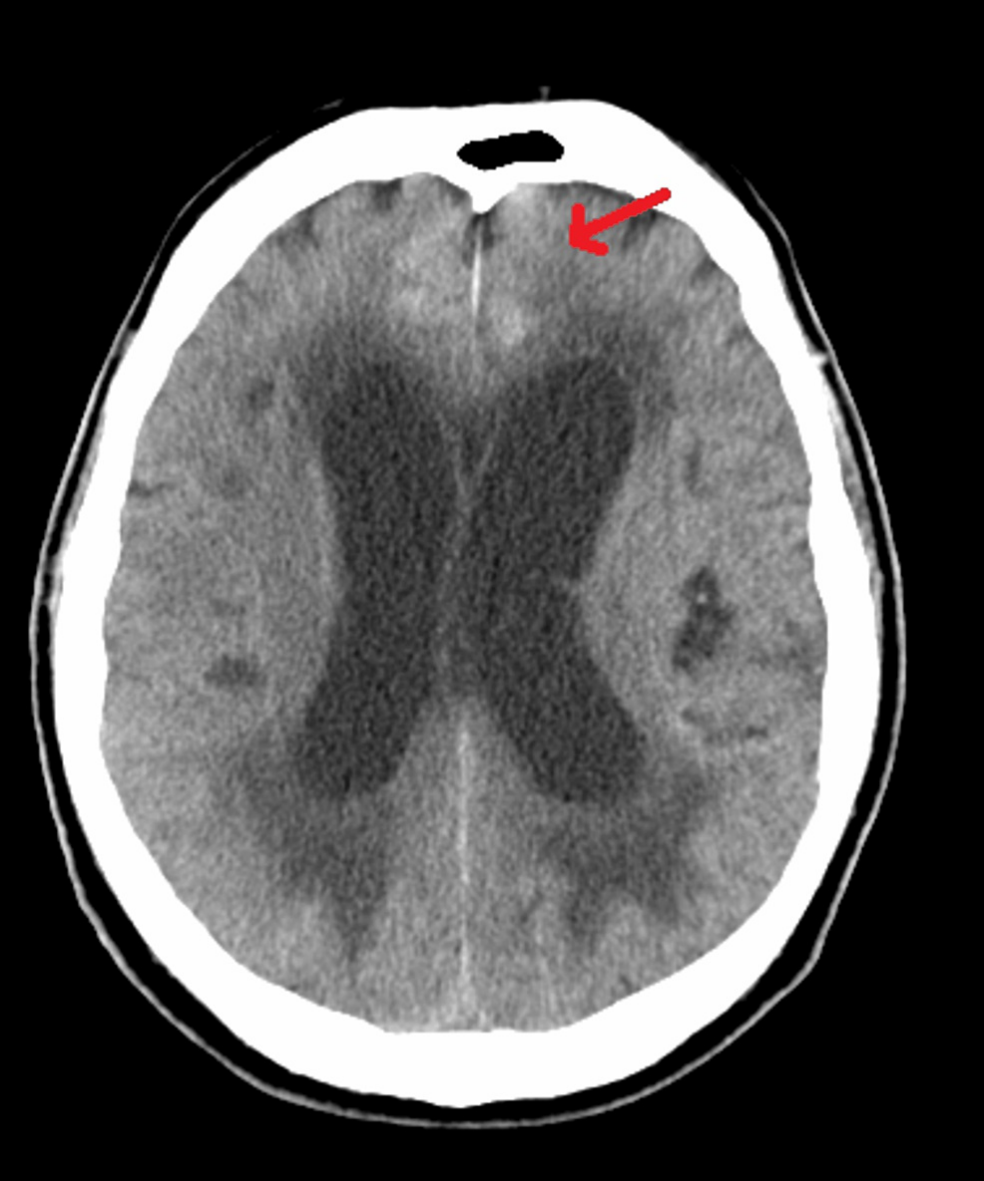
CT of the head without contrast showing subarachnoid hemorrhage above the cortical sulci of the bifrontal lobes with intraventricular extension as well as an interval increase in the hydrocephalus.

A continuous EEG was done and showed mild-moderate generalized slowing. Due to the low CSF glucose and high protein without elevated white cell count in CSF along with the leptomeningeal enhancement, we initiated malignancy workup, which included CT of the chest, abdomen, and pelvis with contrast, which came back negative for primary malignancy or adenopathy. Whole body positron emission tomography (PET) scan came back negative as well. The patient's mental status continued to deteriorate, and the case was discussed with the neurosurgery team, who decided to put on a lumbar drain to relieve the increased intracranial pressure. CSF evaluation from the lumbar drain showed persistent low glucose (31), high protein (64), and normal WBC count (3). The patient's mental status improved immediately after placement of the lumbar drain; however, after two days, the lumbar drain lost patency. The cytology testing from the initial LP came back showing malignant cells and staining confirmed melanoma. An extensive workup was done to look for the primary source, including a dermatology consult for full skin inspection and an ophthalmology consult to look for the ocular source of melanoma; however, no primary source was found. A BRAF mutation test was not done, as the CSF sample was not adequate. The patient’s condition deteriorated quickly after a few days as he developed a subarachnoid hemorrhage above the cortical sulci of the bifrontal lobes with intraventricular extension as well as an interval increase in the hydrocephalus (Figure 6).

Given the poor prognosis and rapidly progressive hydrocephalus, multiple options were discussed with the family, including trying to place a lumbar drain again or a palliative ventriculoperitoneal (VP) shunt; however, due to the presence of subarachnoid hemorrhage with intraventricular extension along with the prior loss of patency of lumbar drain, the family was informed about the high possibility of the lumbar drain or VP shunt blockage. Radiation oncology discussed palliative whole-brain radiation; however, the patient's family decided to pursue palliative measures, and the patient was discharged to home with hospice care.

## Discussion

One of the most devastating complications of any malignancy is the leptomeningeal disease. It is estimated that most leptomeningeal metastasis due to melanoma is underdiagnosed [7].

We present a report of unusual presentation of leptomeningeal metastasis due to melanoma of unknown primary that presented with acute hydrocephalus, which was very difficult to manage due to repeated failure of shunting methods due to obstruction by malignant cells. Initially, the diagnosis of NPH was suspected, given the classic clinical presentation of urinary urgency, gait disturbance, and cognitive decline; however, that was a wrong assumption, given the acuity of the presentation. Unfortunately, there was no brain imaging to compare the first scan to which resulted in confusion at the beginning about the possibility of having NPH. The lumbar puncture was extremely helpful in excluding NPH and it was concerning for malignancy given the low glucose level in CSF and having negative infectious workup. Cytology revealed malignant cells, and markers for melanoma, including monoclonal antibodies to melanocytic cells (HMB-45) and S-100 protein, were helpful in confirming the diagnosis. Despite having a broad workup, the primary source was not found and a presumed primary CNS melanoma diagnosis was made.

The diagnosis of leptomeningeal metastasis is usually missed due to the variety of signs and symptoms that can occur depending on the site of involvement. Risk factors for CNS metastasis in cutaneous melanoma were identified as sex (male), head and neck primary lesion, presence of visceral metastasis (e.g., lung), and ulceration of the primary lesion; however, no risk factors were identified for leptomeningeal metastasis [8].

MRI with gadolinium enhancement for the entire neuraxis remains the study of choice to evaluate patients with suspected leptomeningeal metastasis despite the high false negative rates, which can reach up to 30-60% [9]. The cytology examination of CSF has a sensitivity of 30-50% only for detecting leptomeningeal metastasis [10].

The prognosis of the leptomeningeal disease depends mainly on the primary source; however, leptomeningeal disease due to melanoma is particularly fulminant, with a mean median survival of 10 weeks after the diagnosis [11]. The addition of chemotherapy was studied in a cohort of 110 patients with leptomeningeal metastasis between 1944 and 2002 and the median survival did not change and remained around 10 weeks with only 7% of patients surviving for 12 months [11]. Testing for BRAF, which is the gene encoding the serine-threonine protein kinase, can offer an opportunity for oncogene-targeted therapy like vemurafenib and nivolumab; however, the survival benefit is still unclear [12].

The management of communicating hydrocephalus in cases of leptomeningeal metastasis can be challenging due to the high rate of complications. A case series reviewed ventriculoperitoneal shunting in patients with CNS melanoma and found that all of the patients developed peritoneal metastasis [13]. CSF shunting surgery resulted in rapid improvement in the mental status of 90.3% of patients in a study that was done on 31 patients [14]. The same study found that 6.5% of patients suffered from CNS infection as a complication. There are no studies that evaluated CSF shunting surgery in patients with CNS melanoma. We found that in our patient, CSF shunting surgery is prone to failure due to blockage most likely by malignant cells.

## Conclusions

Acute communicating hydrocephalus should raise the suspicion of malignancy. Despite the recent advancement in the treatment of malignancy and management of hydrocephalus, those interventions are palliative measures for patients with leptomeningeal disease, and they remain to have a poor prognosis.
